# High prevalence of HTLV-1 among blood donors in southeastern Gabon (Central Africa): a major challenge for transfusion safety in a semi-rural area

**DOI:** 10.1128/spectrum.01755-26

**Published:** 2026-08-06

**Authors:** Hanneke Moussonda-Mouélé, Jill-Léa Ramassamy, Eldridge Fedricksen Oloumbou, Antony Idam Mamimandjiami, Noémie Delolme, Christ Eddy Ognari-Ayoumi, Moussa Yaro, Tiburce Etele, Ivan Sosthène Mfouo-Tynga, Olivier Cassar, Antoine Gessain, Augustin Mouinga-Ondémé

**Affiliations:** 1 Unité des Infections Rétrovirales et Pathologies Associées, Centre Interdisciplinaire de Recherches Médicales de Franceville (CIRMF) 89249 , Franceville, Gabon; 2 Unité dʼEpidémiologie et Physiopathologie des Virus Oncogènes, Institut Pasteur, Université de Paris Cité 27058, Paris, France; 3 Département de Biologie, Université des Sciences et Techniques de Masuku 226512, Franceville, Gabon; 4 Service de Laboratoire, Centre Hospitalier Universitaire Amissa Bongo, Franceville, Gabon; Kumamoto Daigaku, Kumamoto, Japan

**Keywords:** HTLV-1, prevalence, blood donors, semi-rural area, Gabon

## Abstract

Sub-Saharan Africa is an endemic region for human T-lymphotropic virus type 1 (HTLV-1). While high prevalence rates have been reported across Gabon, systematic screening of blood donors has not yet been implemented at the national level. A cross-sectional study was conducted on 2,375 blood donations from 2,303 blood donors at the Amissa Bongo Teaching Hospital Center in Franceville. Serological screening was performed via ELISA, followed by Western Blot confirmation and PCR-based proviral DNA amplification of the *env* gene. The overall HTLV-1 seroprevalence was 2.1% (95% CI: 1.6%–2.7%) at the donation level and 1.8% (95% CI: 1.3%–2.5%) at the donor level. The seroprevalence in Franceville was significantly higher than at the national blood bank in Libreville (ORa = 2.52; 95% CI: 1.5–4.2). History of blood transfusion was the only risk factor significantly associated with HTLV-1 infection (OR = 3.73; 95% CI: 1.3–9). Molecular analysis revealed a predominance of the HTLV-1b subtype (32/33), alongside one HTLV-1d strain, a subtype primarily reported in non-human primates. The high HTLV-1 seroprevalence among blood donors in southeastern Gabon, combined with the identification of a history of transfusion as a significant risk factor, represents a major challenge for transfusion safety in a semi-rural area. These results underscore the urgent need to integrate systematic HTLV-1 screening into the transfusion safety algorithm in Gabon to interrupt the cycle of iatrogenic transmission.

**IMPORTANCE:**

Human T-lymphotropic virus type 1 (HTLV-1) remains a major public health problem in Central Africa, with Gabon being a major endemic hotspot. This study addresses a critical issue for blood safety in semi-rural areas, where prevalence in the general adult rural population is exceptionally high, ranging from 7.3% to 8.7% according to previous studies. Here, we report a prevalence of 1.8% among blood donors in southeastern Gabon, a rate 2.5 times higher than that of blood donors in the capital. Moreover, a history of transfusion was identified as the only significant risk factor associated with HTLV-1 infection, directly demonstrating active iatrogenic transmission in the absence of systematic screening. Furthermore, the discovery of the rare HTLV-1d subtype highlights the persistent potential risk of zoonotic and human-to-human transmission in these forest-adjacent ecosystems. These results provide compelling evidence for the urgent integration of HTLV-1 screening in endemic rural areas of Gabon.

## INTRODUCTION

Human T-lymphotropic virus type 1 (HTLV-1), the first oncogenic retrovirus discovered in humans, was isolated in the early 1980s in the United States and in Japan (1, 2). Although frequently overlooked, HTLV-1 remains a significant public health problem, affecting an estimated 5–10 million people worldwide at least (3, 4). Endemic foci are primarily located in southwestern Japan, South America, the Caribbean, Iran, Melanesia, and sub-Saharan Africa (3–5). While most carriers remain asymptomatic throughout their lives, around 2%–7% of infected individuals develop severe clinical conditions. The two primary manifestations are adult T-cell leukemia/lymphoma (ATLL) and tropical spastic paraparesis/HTLV-1-associated myelopathy (HAM/TSP) (5–7).

Central Africa is recognized as one of the primary endemic regions for HTLV-1. Within this region, Gabon occupies a prominent position, with studies indicating very high prevalence rates across the population compared to neighboring countries (8). Notably high prevalence rates have been reported in rural adult populations (7.3%–8.7%), as well as among pregnant women (2.1%) and children (2.8%) (9–12). In contrast, the prevalence is significantly lower in urban centers; among blood donors in Libreville, the rate is approximately 0.7%, likely reflecting the stringent selection criteria for donors (13).

HTLV-1 is transmitted through three primary routes: mother-to-child (especially via prolonged breastfeeding exceeding 6 months), unprotected sexual intercourse, and parental exposure through infected blood (4). The latter is exceptionally efficient; the risk of seroconversion following the transfusion of non-leukocyte-depleted blood products from an HTLV-1-positive donor is estimated to be between 28% and 63% (14, 15). In resource-limited settings, where systematic screening for HTLV-1 is not consistently implemented, blood transfusion remains a significant transmission route that compromises overall transfusion safety (16).

A large-scale survey by Djuicy et al. identified the southeastern region as one with the highest prevalences in the country, approaching 10% among adults in rural settings (9). This hyperendemicity in the general population contrasts sharply with the lack of specific data regarding transfusion safety in the region. Despite high HTLV-1 prevalences in the population, systematic HTLV-1 screening is not currently mandated for blood donors in Gabon. Together, these factors of regional hyperendemicity and the absence of universal screening underscore a potential risk to the blood supply. Consequently, this study aimed to assess the prevalence of HTLV-1 among blood donors in semi-rural areas of Gabon and to assess the resulting iatrogenic transmission risk, while characterizing the molecular diversity of circulating strains.

## MATERIALS AND METHODS

### Blood donors’ enrollment and study design

**Fig 1:**
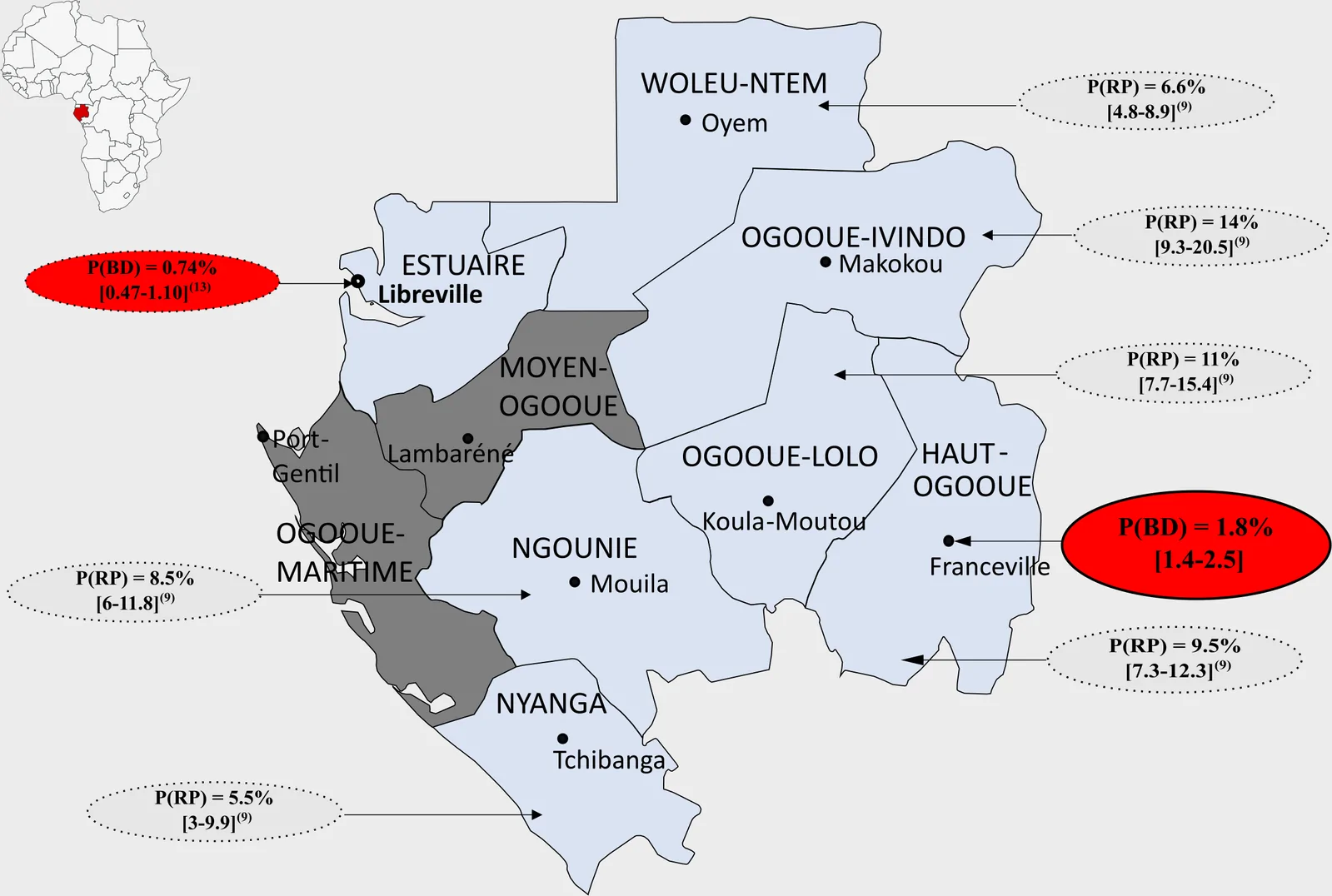
Map of Gabon showing HTLV-1 seroprevalence estimates by study population and location. The seroprevalence among blood donors in Franceville (this study) is indicated by a solid red oval. Previously published estimates are shown for comparison: adult rural population seroprevalence by province in dashed gray ovals (9) and blood donor seroprevalence from the Centre National de Transfusion Sanguine (CNTS) in Libreville in a dashed red oval (13). Provinces in dark gray indicate areas without available rural prevalence data. All estimates are presented with 95% confidence intervals. P(BD): HTLV-1 seroprevalence among blood donors; P(RP): HTLV-1 seroprevalence in the adult rural population. The map was modified from https://commons.wikimedia.org/wiki/Atlas_of_Gabon using Inkscape 1.4.4.

This cross-sectional study was conducted at the Amissa Bongo Teaching Hospital (Centre Hospitalier Universitaire Amissa Bongo; CHUAB) in Franceville, the capital of the Haut-Ogooué province, Gabon (Fig. 1). This facility serves as the sole blood transfusion center for the province. The study population comprised both voluntary and replacement (family) blood donors, enrolled following a preliminary clinical screening. The standard institutional protocol for blood safety included serological screening for HIV, HBV, HCV, and syphilis, as well as testing for microfilariae and determination of ABO and Rhesus blood groups; however, these routine screening results were not available for the purposes of this study. Due to disruptions caused by the COVID-19 pandemic, sample collection was conducted in two distinct phases: an initial period from August 2019 to January 2020, and a second phase from February 2021 to July 2022.

Data were collected, including sociodemographic data (age, gender, and place of birth) and relevant epidemiological history, including history of blood transfusion, unprotected sexual intercourse, prolonged breastfeeding, and donation frequency (first-time or repeat), as well as history of exposure to non-human primates (NHPs), specifically through direct contact or bites.

From each consenting participant, 5–7 mL of whole blood was collected into EDTA tubes. Plasma and buffy coat fractions were isolated within 24–48 h of collection and stored at −80°C until laboratory analysis. A total of 2,684 donations were initially screened; however, 9 were excluded due to inconsistent results (*n* = 2) and missing data (*n* = 7). For risk factor analysis, the data set was restricted to one donation per donor. Duplicate records were identified using a combination of personal identifiers, including first name, surname, sex, age, occupation, and phone number. Duplicate records were classified as either confirmed (identical donor identifiers) or probable (near-matches judged likely to represent the same individual) (Fig. S1). For repeat donors, we retained the earliest donation with complete data.

### Serological tests

All plasma samples were screened for HTLV-1/2 antibodies using a commercially available ELISA kit (HTLV-I/II ELISA 4.0, MP Biomedicals). Samples reactive by ELISA were further analyzed using the HTLV BLOT 2.4 (MP Biomedicals) for confirmatory testing. Results were interpreted according to the manufacturer’s instructions.

### Molecular tests

High molecular weight DNA was extracted from the peripheral blood buffy coats of all ELISA-reactive samples using the QIAamp DNA Blood Mini Kit (Qiagen, Courtaboeuf, France). For detection and characterization of HTLV, a semi-nested polymerase chain reaction (PCR) was performed to amplify a 522 bp fragment of the *env* gene. The reaction utilized Env1/Env22 as the outer primers and Env1/Env2 as the inner primers, following a previously described protocol (17).

### Phylogenetic analysis

The resulting PCR amplicons were sent to Eurofins Genomics (Cologne, Germany) for purification and sequencing using the Sanger method. Initial HTLV-1 genotype identification was performed by comparing the obtained 522 bp *env* sequences against the GenBank database using the BLAST algorithm (http://www.ncbi.nlm.nih.gov/BLAST). Sequences were aligned using MEGA software version 12.1.2, and a phylogenetic tree was inferred using the maximum likelihood (ML) method on Seaview version 4 software.

### Statistical analysis

A donation was classified as HTLV-1 positive if it displayed a definitive WB profile (HTLV-1, HTLV-1/2, or HTLV), or an indeterminate WB profile with a confirmed positive PCR result. The overall seroprevalence of HTLV-1 was estimated at both the donation and donor levels. Seroprevalence estimates were reported with 95% confidence intervals (95% CI). Univariate analyses were performed to assess associations between HTLV-1 seropositivity. Group comparisons were conducted using the chi-square tests or Fisher’s exact test accordingly, and crude odds ratios (ORs) with 95% CIs were assessed using logistic regression. Statistical significance was defined as *P* values <0.05. Data were analyzed using R software (version 4.5.1).

The HTLV-1 prevalence observed among blood donors in this study was compared with previously reported data from rural Gabonese populations and blood donors in Centre National de Transfusion Sanguine (CNTS) Libreville (9, 13). To ensure comparability, these reference studies utilized identical serological and molecular diagnostic criteria; specifically, HTLV-1 infection was defined as positive Western Blot (HTLV-1, HTLV-1/2, or HTLV-undifferentiated profile) or PCR. Finally, adjusted odds ratios (ORa), accounting for age and gender, were calculated to compare the current study with these two reference populations.

## RESULTS

### Serological and molecular results for HTLV-1

A total of 2,675 blood donations were collected from 2,303 enrolled blood donors: 2,009 donors contributed a single donation, and 294 donors contributed multiple donations during the study period (accounting for 666 donations) (Fig. S1). Of the 2,675 plasma samples screened by ELISA, 137 (5.1%) were reactive and subsequently tested by WB. According to the manufacturer’s criteria, 40 samples (29%) showed an HTLV-1 profile, 5 (3.6%) an HTLV-1/2 dual-reactive profile, 8 (5.8%) an undifferentiated HTLV-seroreactive profile, and 42 (31%) an indeterminate profile. All 137 ELISA-reactive samples were further tested by PCR targeting the *env* gene, which yielded successful amplification in 47 samples (34%) (Table 1). Based on the positivity criteria, 56 donations were confirmed HTLV-1 positive, comprising 53 with a reactive WB profile (HTLV-1, HTLV-1/2, and HTLV) and 3 with an indeterminate WB profile and positive PCR. These 56 HTLV-1-positive donations originated from 42 individual donors; the discrepancy reflects the 8 multiple-donation donors who each contributed between 2 and 5 positive donations over the study period. Thus, we obtained an overall donation-level prevalence of 2.1% (95% CI: 1.6%–2.7%). Among the 294 donors who contributed multiple donations during the study period, 8 were HTLV-1 infected and collectively contributed 22 positive donations (2–5 donations per donor), accounting for a seroprevalence of 3.3% among repeat donations (22/666, 95% CI: 2.2%–4.9%).

**TABLE 1:** Western Blot profiles and *env* PCR results among ELISA-reactive blood donations^*a*^

WB profile	*N* (%)	*env* PCR+, *n* (%)
HTLV-1	40 (29)	33/40 (83)
HTLV-1/2	5 (3.6)	5/5 (100)
HTLV	8 (5.8)	6/8 (75)
IND	42 (31)	3/42 (7.1)
NEG	42 (31)	0/42 (0)
Total	137 (100)	47/137 (34)

^*a*^ IND, indeterminate; NEG, negative; WB, Western Blot. *N* (%): number of donations per WB profile (% of total ELISA-reactive samples) and *n *(%): number of PCR-positive samples (positivity rate within each WB profile).

### Study population characteristics

Of the 2,303 donors included in our analysis, 88% (2,018/2,303) were men, and 12% (285/2,303) were women. The median age was 30 years (interquartile range: 25–36). Most participants (77.6%) originated from the Haut-Ogooué province. Based on self-reported donation history, 1,029 donors (44.7%) reported having donated blood prior to the current survey, and 485 (21.1%) were first-time blood donors. Regarding the type of donation, 49.6% (1,143/2,303) were voluntary donors, while 37.4% (861/2,303) were replacement (family) donors (Table 2). In terms of risk factors, a small proportion of participants, 3.6% (82/2,303) reported a history of blood transfusion. The majority of the study population (79.8%, *n* = 1,839) reported a history of unprotected sexual intercourse. Furthermore, while 57.4% of donors reported previous contact with non-human primates, documented cases of bites were rare, affecting only 0.17% of the sample (4/2,303).

**TABLE 2:** HTLV-1 seroprevalence and associated risk factors among 2,303 blood donors^*c*^^,^^*d*^

		HTLV-1 prevalence		Univariate analysis	
Characteristic	*N* (%)	*n* (%) (95% CI)	*P* value^*a*^	Crude OR (95% CI)	*P* value^*b*^
Total	2,303 (100)	42 (1.8) (1.35–2.46)	overall seroprevalence		
Sex			0.7		0.7
M	2,018 (88)	36 (1.8) (1.27–2.49)		1	
F	285 (12)	6 (2.1) (0.86–4.75)		1.18 (0.45–2.64)	
Age category			0.3		0.3
<25	655 (28)	11 (1.7) (0.89–3.08)		1	
25–35	1,060 (46)	16 (1.5) (0.90–2.50)		0.9 (0.42–2)	
>35	588 (26)	15 (2.6) (1.49–4.27)		1.53 (0.7–3.45)	
Period of inclusion			>0.9		>0.9
Aug. 2019 to Jan. 2020	527 (23)	10 (1.9) (0.97–3.58)		1	
Feb. 2021 to July 2021	804 (35)	15 (1.9) (1.09–3.13)		0.98 (0.44–2.28)	
Aug. 2021 to Jan. 2022	787 (34)	13 (1.7) (0.92–2.88)		0.87 (0.38–2.05)	
Feb. 2022 to July 2022	185 (8.0)	4 (2.2) (0.69–5.80)		1.14 (0.31–3.46)	
History of blood donation			>0.9		0.9
Repeat donor	1,029 (45)	18 (1.7) (1.07–2.81)		1	
First-time donor	485 (21)	9 (1.9) (0.91–3.62)		1.06 (0.45–2.32)	
NA	789 (34)	15 (1.9) (1.11–3.19)		–^*e*^	
Type of blood donors			0.8		>0.9
Volunteer donor	1,143 (50)	22 (1.9) (1.24–2.95)		1	
Familial donor	861 (37)	16 (1.9) (1.10–3.07)		0.96 (0.5–1.84)	
NA	299 (13)	4 (1.3) (0.43–3.62)		–	
History of transfusion			**0.033**		**0.021**
No	1,931 (84)	33 (1.7) (1.20–2.42)		1	
Yes	82 (3.6)	5 (6.1) (2.27–14.3)		3.73 (1.25–9.04)	
NA	290 (13)	4 (1.4) (0.44–3.73)		–	
Prolonged breast-feeding			0.6		0.7
No	473 (21)	8 (1.7) (0.79–3.44)		1	
Yes	1,505 (65)	30 (2.0) (1.37–2.87)		1.18 (0.56–2.78)	
NA	325 (14)	4 (1.2) (0.39–3.34)		–	
Unprotected sexual intercourse			0.8		0.4
No	172 (7.5)	2 (1.2) (0.20–4.58)		1	
Yes	1,839 (80)	36 (2.0) (1.39–2.73)		1.7 (0.51–10.5)	
NA	292 (13)	4 (1.4) (0.44–3.71)		–	
Contact with an NHP			0.6		0.5
No	686 (30)	11 (1.6) (0.85–2.94)		1	
Yes	1,323 (57)	27 (2.0) (1.38–3.00)		1.28 (0.65–2.7)	
NA	294 (13)	4 (1.4) (0.44–3.68)		–	
Bitten by an NHP			>0.9		ND
No	871 (38)	16 (1.8) (1.09–3.03)		–	
Yes	4 (0.2)	0 (0) (0–60.4)		–	
NA	1,428 (62)	26 (1.8) (1.22–2.70)		–	

^*a*^ *P* value from chi-square or Fisher’s exact tests.

^*b*^ *P* value from univariate logistic regression excluding donors with missing data.

^*c*^ *N* (%): number of blood donors in each category (% of total) and *n* (%) (95% CI): number of HTLV-1 seropositive donors (%) (seroprevalence with 95% CI). Bold *P* values indicate statistical significance (*P *< 0.05).

^*d*^ CI, confidence interval; OR, odds ratio; NA, not available; ND, not determined; NHP, non-human primate.

^*e*^ "–” not determined.

### Phylogenetic analysis

**Fig 2:**
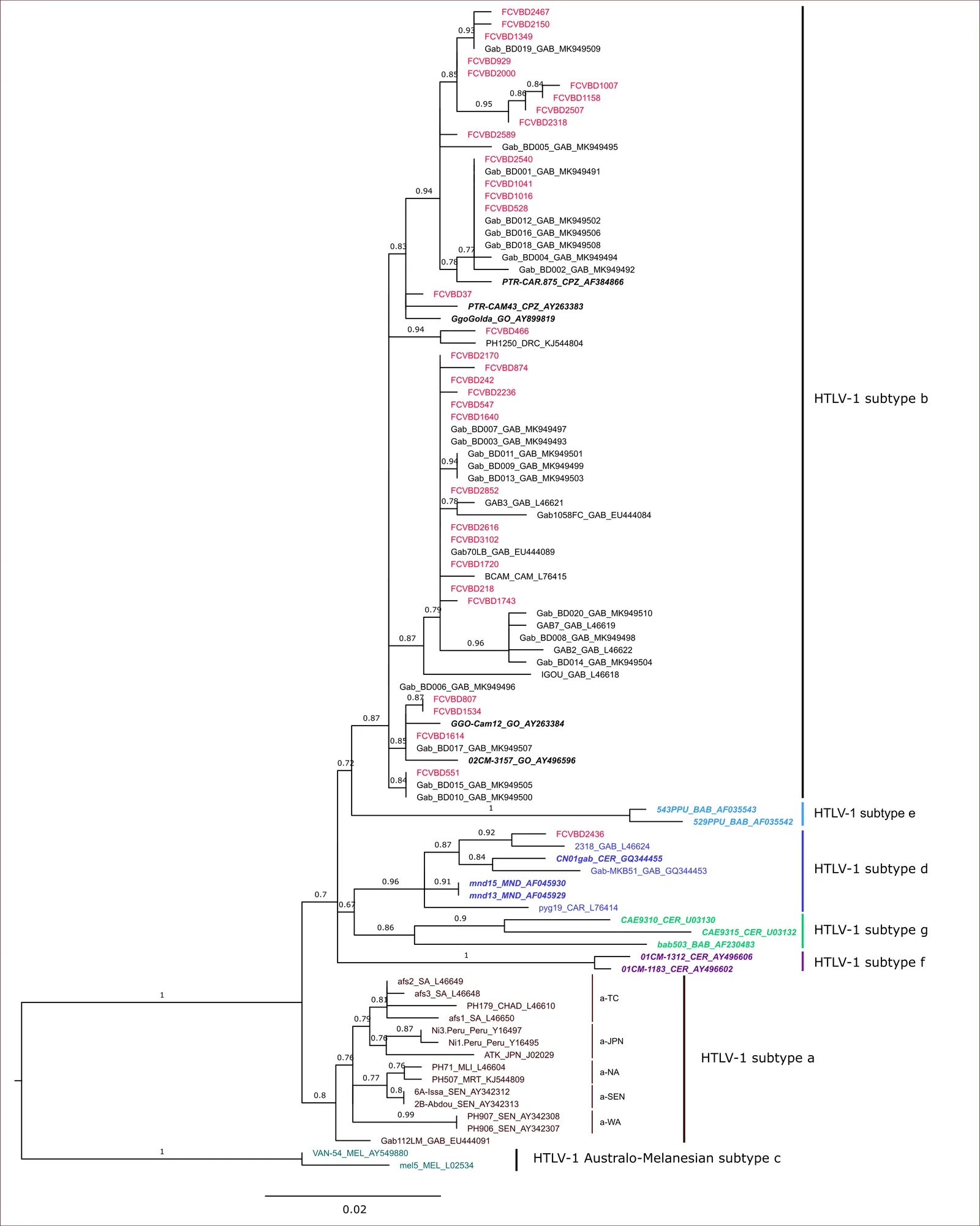
Phylogenetic tree of the *env* gene constructed using the maximum likelihood method based on a 522 bp fragment. Phylogenetic comparisons were performed using a 522-nucleotide fragment of the *env* gp21 gene, obtained from 85 HTLV-1 isolates, including 33 new sequences (in red) and 52 previously published strains, among which 20 sequences were derived from the study of blood donors in Libreville (Gab_BD), 3 from pregnant women in Gabon (Gab), and 29 reference strains. The STLV-1 sequences are indicated in bold and italics. a-TC, a-JPN, a-SEN, a-WA, and a-NA correspond, respectively, to the transcontinental, Japanese, Senegalese, West African, and North African clades of genotype a. The phylogeny was constructed using the ML method with the GTR model. Branch lengths are drawn to scale, with the bar representing 0.01 nucleotide substitutions per site. Maximum posterior probabilities were calculated and plotted on the maximum likelihood tree (threshold value ≥0.50). The GenBank accession numbers for the new sequences from infected blood donors are PZ113065–PZ113097.

From the 42 positive blood donors, 33 strains could be characterized. BLAST and phylogenetic analysis of the 33 characterized strains, based on the 522 bp segment of the *env* gene, revealed that 32 of the isolates belonged to the Central African predominant HTLV-1b subtype, and 1 strain was identified as HTLV-1d subtype. Nucleotide divergence assessment showed a certain degree of diversity among HTLV-1b strains, ranging from 0.2% to 2.8%. The HTLV-1d subtype strain exhibited a slight divergence of 0.96% from the reference strain HL12318G (accession number: L46624), which was previously described and characterized in the no Pygmy population living east of Gabon (17) (Fig. 2).

### Epidemiological analysis

**Fig 3:**
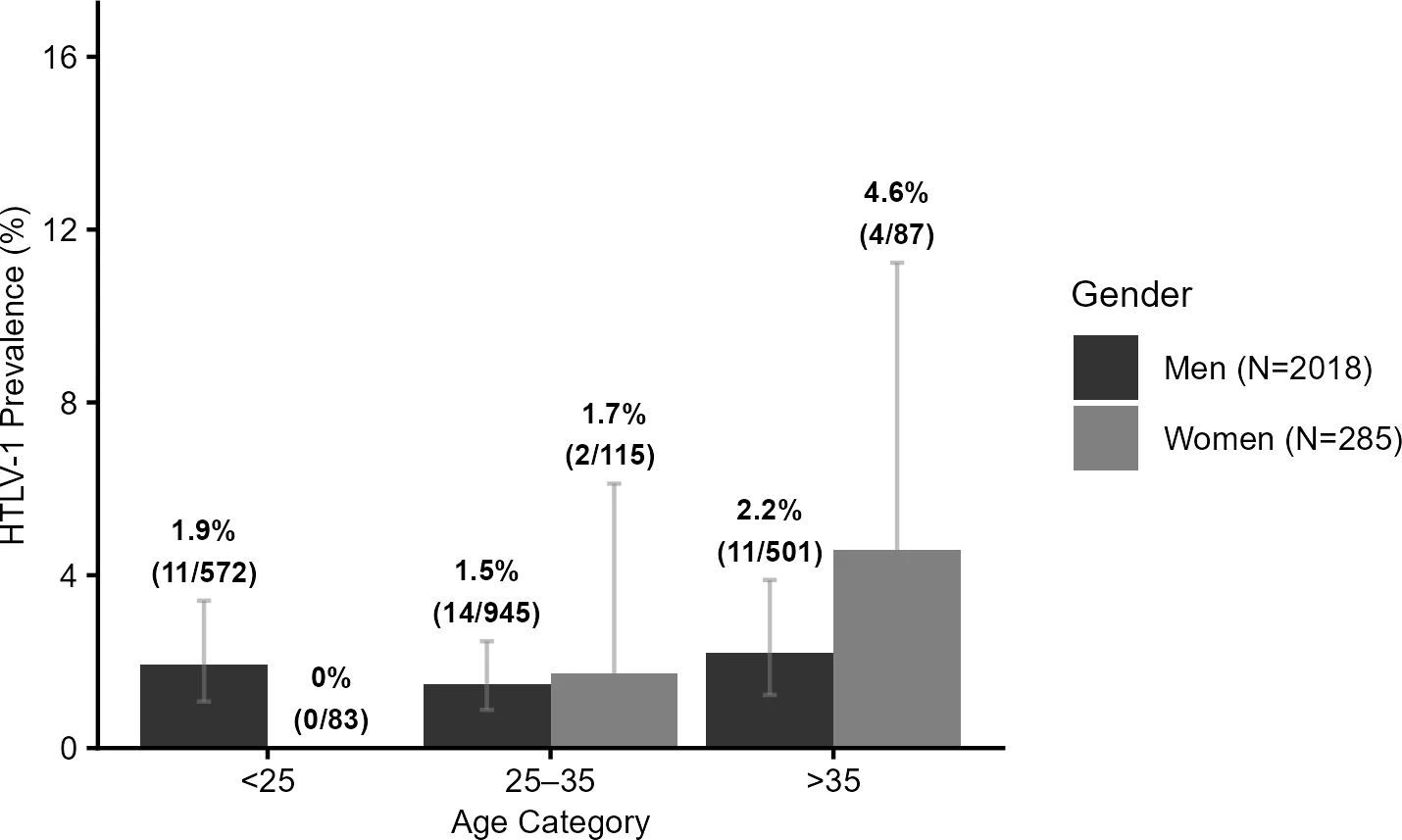
HTLV-1 seroprevalence among 2,303 blood donors stratified by age category and sex. Bars represent seroprevalence estimates with 95% confidence intervals calculated using the Wilson method. Values above bars indicate seroprevalence (%) with the corresponding number of positive donors over the total number of donors tested in each category (*n*/*N*).

At the donor level, 42 of the 2,303 blood donors were diagnosed as HTLV-1 positive, representing an overall prevalence of 1.8% (CI: 1.35–2.46). Analysis of sociodemographic data revealed no significant differences in prevalence based on gender and age. Although a trend toward increased prevalence with age was observed, reaching 2.6% among donors aged over 36 years (2.2% in men and 4.6% in women) compared to 1.7% among those under 25 years, this difference did not reach statistical significance (*P* = 0.30) (Fig. 3; Table 2). Among the potential risk factors evaluated, a history of blood transfusion was the only variable significantly associated with HTLV-1 infection in our study. The risk of HTLV-1 infection was nearly four times higher among previous transfusion recipients (OR = 3.73; 95% CI: 1.25–9.04) (Table 2).

To evaluate the epidemiological specificities of blood donors in Franceville, our data were compared with two previously published studies in Gabon: blood donors from the CNTS in the capital city, Libreville (*N* = 3,123) and a general rural population (*N* = 2,060) (9, 13).

**Fig 4:**
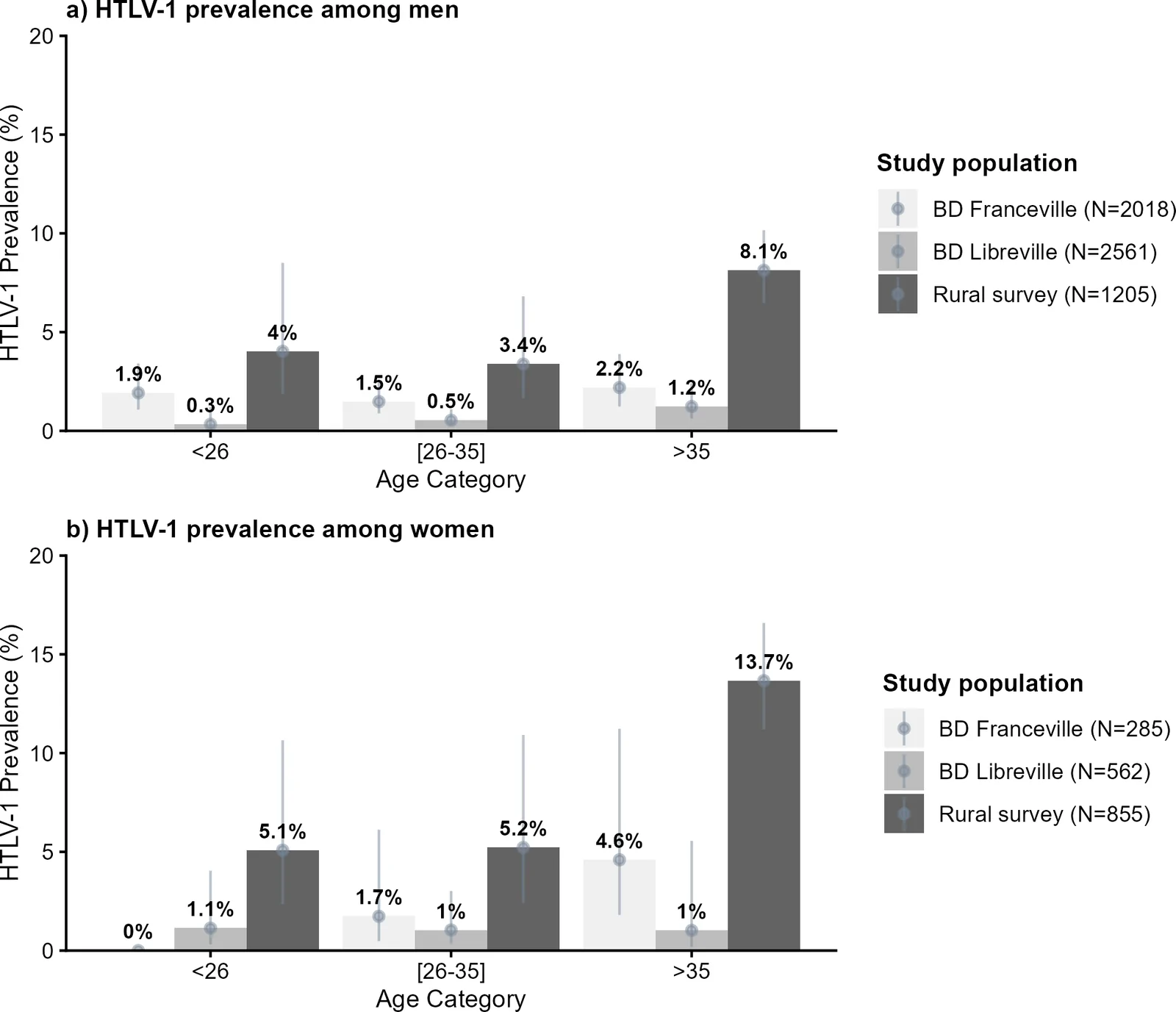
HTLV-1 seroprevalence stratified by sex, age category, and study population. Panel **a** shows estimates for men, and panel **b** shows estimates for women. Three populations are compared: blood donors from Franceville (this study, light gray), blood donors from the Centre National de Transfusion Sanguine (CNTS) in Libreville (gray) (13), and a rural population survey (dark gray) (9). Bars represent 95% confidence intervals calculated using the Wilson method. BD: blood donors and *N* = total number of individuals tested in each category.

ORa were calculated separately for each reference group, with adjustment for sex and age category, given substantial differences in demographic composition between populations (Table 3). Although the donor populations in Libreville and Franceville share similar sociodemographic characteristics, the prevalence of HTLV-1 was significantly higher in Franceville (1.8%; 95% CI: 1.3–2.5) than in Libreville (0.74%; 95% CI: 0.47–1.10) (13). After adjustment for age and gender, blood donors in Franceville carried a 2.5 times higher risk of infection than their counterparts in Libreville (ORa = 2.52; CI 95%: 1.5–4.2), and considerably lower than the overall rural population (ORa = 0.32; 95% CI: 0.2–0.5) where prevalence was 8.7% (95% CI: 7.5–10.0) (9) (Table 2; Fig. 4).

**TABLE 3:** Comparison of HTLV-1 prevalence and seroprevalence estimates across three populations in Gabon^*a*^^*b*^

	Blood donors Franceville (*N* = 2,303)	Blood donors CNTS Libreville (*N* = 3,123)	Rural population (*N* = 2,060)
Men, *n* (%)	2,018 (88)	2,561 (82)	1,205 (58)
Age, mean (range)	30.9 (16–88)	30.9 (17–59)	49.1 (15–98)
HTLV-1 *n*+/*N*	42/2,303	23/3,123	179/2,060
HTLV-1 prevalence (95% CI)	1.8% (1.3–2.5)	0.7% (0.47–1.10)	8.7% (7.5–10)
ORa vs CNTS Libreville (95% CI)	2.52 (1.5–4.2)	1	–
ORa vs rural population (95% CI)	0.32 (0.2–0.5)	–	1

^*a*^ *N* = total number of individuals tested, *n *(%) = number of men tested, *n*+ = number of HTLV-1 infected individuals, CI = confidence interval, ORa = adjusted odds ratios, CNTS = Centre National de Transfusion Sanguine.

^*b*^ "–” not determined.

## DISCUSSION

HTLV-1 infection remains a significant yet largely overlooked public health challenge, particularly in endemic regions like Central Africa (16). Gabon carries one of the highest HTLV-1 prevalences recorded in adult populations worldwide (9). Our study conducted among blood donors in Franceville, a semi-rural city in southeastern Gabon, reveals an association that suggests a potential risk of iatrogenic HTLV-1 transmission through blood transfusion. We observed an overall prevalence among blood donations of 2.1% (95% CI: 1.6%–2.7%). The donor-level prevalence was 1.8% (95% CI: 1.35%–2.46%), 2.5 times higher than that previously reported at the National Blood Transfusion Center (CNTS) in the capital city Libreville (0.7%) (13). These figures stand in stark contrast to HTLV-1 prevalence reported in other African blood banks, where significantly lower rates have been documented: 0.062% in South Africa (*N* = 46,716 donors), 0.16% in Senegal (*N* = 4,900), 0.19% in Ethiopia (*N* = 1,600), and 0.89% in Mozambique (*N* = 2,019) (18–21).

The elevated prevalence observed in Franceville blood donors directly reflects the hyperendemicity of HTLV-1 in the general rural adult population of southeastern Gabon, estimated at 9.5% in the Haut-Ogooué province (9). That our donor-level prevalence (1.8%) remains substantially lower than this general population estimate is expected and largely attributable to the well-described “healthy donor effect.” Indeed, donors constitute a highly preselected subpopulation, predominantly young and male (88% in our study), which structurally underrepresents the demographic groups carrying the highest HTLV-1 burden (particularly older women).

Yet, even within this preselected population, the observed seroprevalence carries significant transfusion safety implications, directly supported by risk factors analysis identifying a history of blood transfusion as the only predictor of HTLV-1 infection (OR = 3.73; 95% CI: 1.25–9.04). Nosocomial transmission of HTLV-1 has been previously documented across Central Africa (9, 22–24). In rural Gabon and Cameroon, history of transfusion and number of blood units received were identified as risk factors in univariate analyses, although multivariate analyses in these studies highlighted history of hospitalization and surgery as the strongest independent predictors, collectively pointing to a broader nosocomial transmission pattern (9, 23). Similarly, in the DRC and CAR, blood transfusion and parenteral injections have been identified as significant vehicles of HTLV-1 spread (22, 24). This is of particular concern given that the risk of seroconversion following transfusion of HTLV-1-infected blood components is extremely high, ranging from 28% to 63% (15). Unlike high-income and endemic regions such as Japan, France, and parts of the United States, where systematic HTLV-1 screening of blood donors has been implemented, no such routine screening exists in Gabonese hospitals despite the high prevalence in the rural population. This diagnostic gap has consequences for highly transfusion-dependent and vulnerable populations, particularly children with sickle cell disease (drepanocytosis) and women experiencing obstetric complications. Previous studies conducted in Franceville have demonstrated that 19.2% of children with sickle cell disease were infected with HTLV-1, compared to only 1% in the general pediatric population (11). This stark disparity highlights the profound impact of unscreened blood transfusions on the long-term health of chronic patients in the region.

Although the majority of HTLV-1 carriers remain asymptomatic, approximately 2%–7% of them will develop ATLL or HAM/TSP during their lifetime, conditions that are debilitating and often fatal (25). A critical aspect of transfusion risk is that blood-borne transmission of the virus could increase the risk of developing HAM/TSP with a rapid onset (18, 26). In Gabon, these diseases are chronically underreported, reflecting the broader systemic neglect of this retrovirus (27). Recent hospital-based data from Libreville underscore this clinical reality: of 352 patients, 7.39% (26/352) were confirmed HTLV-1 carriers. Critically, among those presenting with neurological symptoms, 55.6% (5/9) were diagnosed with TSP/HAM (28). These findings underscore the urgent need for systematic HTLV-1 screening to prevent further iatrogenic spread of a retrovirus responsible for severely debilitating and irreversible neurological disease. In Japan, the introduction of universal screening has successfully reduced the number of HTLV-1 carriers by approximately 40% over the last few decades (29). While financial constraints are frequently cited as barriers to screening implementation in resource-limited settings, these initial costs must be weighed against the long-term medical and socioeconomic burden of HTLV-1-associated diseases. Cost-effectiveness analyses conducted in other endemic regions, such as Japan and Brazil, have demonstrated that both prenatal and transfusion-based screening programs can prevent cases of ATLL and TSP/HAM, ultimately reducing mortality and preserving hospital resources in the long term (30, 31). Conversely, an economic assessment in South Africa did not support systematic screening, largely driven by low prevalence in that setting (32), an argument that would not apply to a hyperendemic region such as southeastern Gabon, where the absence of HTLV-1 screening has direct consequences for transfusion safety, notably in obstetric and pediatric emergency settings.

While leukoreduction or prolonged storage (>10 days) can mitigate the transmission of this cell-associated virus (15, 33), these supplemental measures cannot substitute for systematic serological screening and molecular confirmation. Handling indeterminate serological profiles represents a significant challenge for transfusion safety in endemic areas, as it requires the systematic use of confirmatory tests (INNO-LIA, PCR), which are essential given the high rate of false positives (34, 35). These confirmatory tests are extremely costly, making them difficult to incorporate into public health policies (18). The discrepancy observed between initial screening and molecular confirmation highlights the limitations of current algorithms: the exclusion of these profiles, although essential to prevent iatrogenic risk, leads to the unjustified rejection of healthy donors and depletes blood supplies. These competing constraints highlight the need for locally adapted transfusion algorithms that can reconcile donor safety, blood supply adequacy, and the limited availability of confirmatory testing in endemic resource-limited settings.

Phylogenetic analysis of the 33 characterized strains reveals a near-exclusive predominance of the HTLV-1b subtype (32/33), consistent with previous molecular epidemiological studies, confirming genotype b as the major circulating strain in Central Africa (8, 10, 12, 13). Interestingly, we identified one strain belonging to the divergent HTLV-1d subtype, a rare genotype phylogenetically closely related to strains of simian T-lymphotropic virus type 1 (STLV-1) circulating among NHPs in Central African tropical forests, and typically associated with zoonotic transmission through NHP bites (17, 36). Although the donor carrying this strain reported contact with NHPs, no bite was documented, precluding definitive attribution to either direct zoonotic transmission or to secondary human-to-human transmission, as previously described for this subtype in Cameroon (37). To date, HTLV-1d remains a rare subtype, representing up to 3% of HTLV-1 infections in Central Africa (38). Clear evidence of its full adaptation to the human host or of its specific clinical pathogenicity is lacking. These observations underscore the need for further studies to determine the respective roles of zoonotic events and human-to-human transmission routes in the spread of different HTLV-1 genotypes.

This study has several limitations. First, the epidemiological analysis was limited by missing data and the absence of specific variables, such as the number of sexual partners, duration of breastfeeding, and granular details regarding NHP hunting and butchering practices, preventing robust multivariate analysis of factors that are otherwise well-established routes of HTLV-1 transmission. Furthermore, there is an evident selection bias, as our blood donor population is predominantly young and male, which does not adequately represent the demographic structure of the general population in the region, consequently limiting the generalizability of our prevalence estimates. Moreover, the study is subject to recall bias, as data relied exclusively on participants’ self-reports. Second, although the history of transfusion was identified as a significant risk factor, the absence of data on transfusion indication prevented further characterization of the contexts in which iatrogenic transmission most likely occurred.

### Conclusion

Our study shows that southeastern Gabon has one of the highest reported prevalences of HTLV-1 among blood donors on the African continent. The identification of a history of transfusion as a significant risk factor highlights a critical vulnerability in blood safety within endemic semi-rural areas. This high risk of iatrogenic transmission makes it imperative to integrate systematic HTLV-1 screening into Gabon’s national blood safety algorithm. Such a measure is an essential step toward breaking the cycle of viral transmission and reducing the long-term clinical burden of this neglected retroviral infection.

## Data Availability

Available from the corresponding author on reasonable request.

## SUPPLEMENTAL MATERIAL

Figure S1 spectrum.01755-26-s0001.tiff Duplicates were defined as multiple records for the same blood donors (confirmed) or near matches judged likely to be the same blood donors (probable). For repeat donors, we retained one donation with complete data; if multiple were complete, we retained the earliest donation.

Supplemental legend spectrum.01755-26-s0002.docx Legend for Fig. S1.

ASM does not own the copyrights to Supplemental Material that may be linked to, or accessed through, an article. The authors have granted ASM a non-exclusive, world-wide license to publish the Supplemental Material files. Please contact the corresponding author directly for reuse.

## References

[1] Yoshida M, Miyoshi I, Hinuma Y. 1982. Isolation and characterization of retrovirus from cell lines of human adult T-cell leukemia and its implication in the disease. Proc Natl Acad Sci USA 79:2031–2035. doi:10.1073/pnas.79.6.2031 6979048 PMC346116

[2] Poiesz BJ, Ruscetti FW, Gazdar AF, Bunn PA, Minna JD, Gallo RC. 1980. Detection and isolation of type C retrovirus particles from fresh and cultured lymphocytes of a patient with cutaneous T-cell lymphoma. Proc Natl Acad Sci USA 77:7415–7419. doi:10.1073/pnas.77.12.7415 6261256 PMC350514

[3] WHO. 2021. Human T-lymphotropic virus type 1: technical report 1st. World Health Organization. Geneva

[4] Gessain A, Cassar O. 2012. Epidemiological aspects and world distribution of HTLV-1 infection. Front Microbiol 3. doi:10.3389/fmicb.2012.00388 PMC3498738 23162541

[5] Branda F, Romano C, Pavia G, Bilotta V, Locci C, Azzena I, Deplano I, Pascale N, Perra M, Giovanetti M, Ciccozzi A, De Vito A, Quirino A, Marascio N, Matera G, Madeddu G, Casu M, Sanna D, Ceccarelli G, Ciccozzi M, Scarpa F. 2025. Human T-lymphotropic virus (HTLV): epidemiology, genetic, pathogenesis, and future challenges. Viruses 17:664. doi:10.3390/v17050664 40431676 PMC12115942

[6] Uchiyama T. 1997. Human T cell leukemia virus type I (HTLV-I) and human diseases. Annu Rev Immunol 15:15–37. doi:10.1146/annurev.immunol.15.1.15 9143680

[7] Gessain A, Vernant JC, Maurs L, Barin F, Gout O, Calender A, De Thé G. 1985. Antibodies to human T-lymphotropic virus type-I in patients with tropical spastic paraparesis. Lancet 326:407–410. doi:10.1016/S0140-6736(85)92734-5 2863442

[8] Gessain A, Ramassamy JL, Afonso PV, Cassar O. 2023. Geographic distribution, clinical epidemiology and genetic diversity of the human oncogenic retrovirus HTLV-1 in Africa, the world’s largest endemic area. Front Immunol 14:1043600. doi:10.3389/fimmu.2023.1043600 36817417 PMC9935834

[9] Djuicy DD, Mouinga-Ondémé A, Cassar O, Ramassamy J-L, Idam Mamimandjiami A, Bikangui R, Fontanet A, Gessain A. 2018. Risk factors for HTLV-1 infection in Central Africa: a rural population-based survey in Gabon. PLoS Negl Trop Dis 12:e0006832. doi:10.1371/journal.pntd.0006832 30312301 PMC6200283

[10] Etenna S-D, Caron M, Besson G, Makuwa M, Gessain A, Mahé A, Kazanji M. 2008. New insights into prevalence, genetic diversity, and proviral load of human T-cell leukemia virus types 1 and 2 in pregnant women in Gabon in equatorial central Africa. J Clin Microbiol 46:3607–3614. doi:10.1128/JCM.01249-08 18845819 PMC2576568

[11] Delaporte E, Peeters M, Bardy JL, Ville Y, Placca L, Bedjabaga I, Larouzé B, Piot P. 1988. Blood transfusion as a major risk factor for HTLV-I infection among hospitalized children in Gabon (Equatorial Africa). J Acquir Immune Defic Syndr 6:424–428. 8455148

[12] Caron M, Besson G, Padilla C, Makuwa M, Nkoghe D, Leroy E, Kazanji M. 2018. Revisiting human T-cell lymphotropic virus types 1 and 2 infections among rural population in Gabon, central Africa thirty years after the first analysis. PLoS Negl Trop Dis 12:e0006833. doi:10.1371/journal.pntd.0006833 30359373 PMC6201875

[13] Ramassamy J-L, Cassar O, Toumbiri M, Diané A, Idam Mamimandjiami A, Bengone C, Ntsame-Ndong JM, Mouinga-Ondémé A, Gessain A. 2020. High prevalence of human T-cell leukemia virus type-1b genotype among blood donors in Gabon, Central Africa. Transfusion 60:1483–1491. doi:10.1111/trf.15838 32415686 PMC7496943

[14] Manns A, Wilks RJ, Murphy EL, Haynes G, Figueroa JP, Barnett M, Hanchard B, Blattner WA. 1992. A prospective study of transmission by transfusion of HTLV‐I and risk factors associated with seroconversion. Int J Cancer 51:886–891. doi:10.1002/ijc.2910510609 1639536

[15] Murphy EL. 2016. Infection with human T-lymphotropic virus types-1 and -2 (HTLV-1 and -2): Implications for blood transfusion safety. Transfus Clin Biol 23:13–19. doi:10.1016/j.tracli.2015.12.001 26778839 PMC5042452

[16] Twizere J-C, Rosadas C, Kidiga M, Djalo F, Murphy EL, Mouinga-Ondeme A, Vermeulen M, Morrell L, Diop S, Maseko S, Sebulime P, Agbleke AA, Fundisi B, Umumararungu E, Watber P, Hlela C, Nhavene E, Chidarikire T, Hermine O, Gessain A. 2025. Strengthening awareness and response to HTLV-1 infection in Africa: a neglected threat to blood safety and public health. Hemasphere 9:e70234. doi:10.1002/hem3.70234 41064106 PMC12501605

[17] Mahieux R, Ibrahim F, Mauclere P, Herve V, Michel P, Tekaia F, Chappey C, Garin B, Van Der Ryst E, Guillemain B, Ledru E, Delaporte E, de The G, Gessain A. 1997. Molecular epidemiology of 58 new African human T-cell leukemia virus type 1 (HTLV-1) strains: identification of a new and distinct HTLV-1 molecular subtype in Central Africa and in Pygmies. J Virol 71:1317–1333. doi:10.1128/JVI.71.2.1317-1333.1997 8995656 PMC191187

[18] Vermeulen M, Sykes W, Coleman C, Custer B, Jacobs G, Jaza J, Kaidarova Z, Hlela C, Gessain A, Cassar O, Poole C, Ingram C, Murphy EL, Reddy R. 2019. The prevalence of human T-lymphotropic virus type 1 & 2 (HTLV-1/2) in South African blood donors. Vox Sang 114:451–458. doi:10.1111/vox.12778 30950074 PMC7413069

[19] Diop S, Calattini S, Abah-Dakou J, Thiam D, Diakhaté L, Gessain A. 2006. Seroprevalence and molecular epidemiology of human T-cell leukemia virus type 1 (HTLV-1) and HTLV-2 in blood donors from Dakar, Senegal. J Clin Microbiol 44:1550–1554. doi:10.1128/JCM.44.4.1550-1554.2006 16597891 PMC1448682

[20] Vrielink H, Sisay Y, Reesink HW, Woerdeman M, Winkel C, de Leeuw SJ, Lelie PN, van der Poel CL. 1995. Evaluation of a combined lysate/recombinant antigen anti-HTLV-I/II ELISA in high and low endemic areas of HTLV-I/II infection. Transfus Med 5:135–137. doi:10.1111/j.1365-3148.1995.tb00201.x 7655577

[21] Gudo ES, Abreu CM, Mussá T, Augusto A do R, Otsuki K, Chambo E, Amade N, Tanuri A, Ferreira OC Jr, Jani IV, HTLV in Mozambique Study Group. 2009. Serologic and molecular typing of human T-lymphotropic virus among blood donors in Maputo City, Mozambique. Transfusion 49:1146–1150. doi:10.1111/j.1537-2995.2009.02100.x 19222818

[22] Pépin J, Labbé A-C, Mamadou-Yaya F, Mbélesso P, Mbadingaï S, Deslandes S, Locas M-C, Frost E. 2010. Iatrogenic transmission of human T cell lymphotropic virus type 1 and hepatitis C virus through parenteral treatment and chemoprophylaxis of sleeping sickness in colonial Equatorial Africa. Clin Infect Dis 51:777–784. doi:10.1086/656232 20735238

[23] Ramassamy J-L, Bilounga Ndongo C, Nnuka P, Antunes M, Le Mener M, Betsem A Betsem E, Njouom R, Cassar O, Fontanet A, Gessain A. 2023. Epidemiological evidence of nosocomial and zoonotic transmission of human T-cell leukemia virus-1 in a large survey in a rural population of Central Africa. J Infect Dis 227:752–760. doi:10.1093/infdis/jiac312 35867855 PMC10043981

[24] Hogan CA, Iles J, Frost EH, Giroux G, Cassar O, Gessain A, Dion M-J, Ilunga V, Rambaut A, Yengo-Ki-Ngimbi A-É, Behets F, Pybus OG, Pépin J. 2016. Epidemic history and iatrogenic transmission of blood-borne viruses in mid-20th century Kinshasa. J Infect Dis 214:353–360. doi:10.1093/infdis/jiw009 26768251

[25] Mardi S, Rashidian M, Bastan F, Molaverdi G, Mozhgani SH. 2025. The role of human leukocyte antigen in HTLV-1 infection and progression to ATLL and HAM/TSP: a systematic review and meta-analysis. Virol J 22:13. doi:10.1186/s12985-024-02612-7 39833815 PMC11749399

[26] Gout O, Baulac M, Gessain A, Semah F, Saal F, Périès J, Cabrol C, Foucault-Fretz C, Laplane D, Sigaux F. 1990. Rapid development of myelopathy after HTLV-I infection acquired by transfusion during cardiac transplantation. N Engl J Med 322:383–388. doi:10.1056/NEJM199002083220607 2300089

[27] Martin F, Tagaya Y, Gallo R. 2018. Time to eradicate HTLV-1: an open letter to WHO. Lancet 391:1893–1894. doi:10.1016/S0140-6736(18)30974-7 29781438

[28] Oloumbou EF, Ledaga Lentombo L, Obame-Nkoghe J, Iba-Ba J, Engone Ondo JD, Ognari Ayoumi C, Yaro M, Diané A, Kouna Ndouongo P, Boguikouma J-B, Mfouo-Tynga I, Mouinga-Ondémé A. 2025. Clinical and molecular evidences of HTLV-1 infection in inpatients diagnosed with diseases previously described as associated to this infection: a case series in Gabon, Central Africa. PLoS Negl Trop Dis 19:e0013075. doi:10.1371/journal.pntd.0013075 40367287 PMC12101779

[29] Satake M, Sagara Y, Hamaguchi I. 2023. Lower prevalence of anti-HTLV-1 as expected by previous models among first-time blood donors in Japan. J Med Virol 95:e28606. doi:10.1002/jmv.28606 36815496

[30] Kowada A. 2023. Cost-effectiveness of human T-cell leukemia virus type 1 (HTLV-1) antenatal screening for prevention of mother-to-child transmission. PLoS Negl Trop Dis 17:e0011129. doi:10.1371/journal.pntd.0011129 36809372 PMC9983854

[31] Rosadas C, Senna K, da Costa M, Assone T, Casseb J, Nukui Y, Cook L, Mariano L, Galvão Castro B, Rios Grassi MF, Penalva de Oliveira AC, Caterino-de-Araujo A, Malik B, Boa-Sorte N, Peixoto P, Puccioni-Sohler M, Santos M, Taylor GP. 2023. Economic analysis of antenatal screening for human T-cell lymphotropic virus type 1 in Brazil: an open access cost-utility model. Lancet Glob Health 11:e781–e790. doi:10.1016/S2214-109X(23)00065-7 37061315

[32] Vermeulen M, van den Berg K, Sykes W, Reddy R, Ingram C, Poole C, Custer B. 2019. Health economic implications of testing blood donors in South Africa for HTLV 1 & 2 infection. Vox Sang 114:467–477. doi:10.1111/vox.12788 31131453 PMC7370940

[33] Hewitt PE, Davison K, Howell DR, Taylor GP. 2013. Human T-lymphotropic virus lookback in NHS Blood and Transplant (England) reveals the efficacy of leukoreduction. Transfusion 53:2168–2175. doi:10.1111/trf.12105 23384161

[34] Filippone C, Bassot S, Betsem E, Tortevoye P, Guillotte M, Mercereau-Puijalon O, Plancoulaine S, Calattini S, Gessain A. 2012. A new and frequent human T-cell leukemia virus indeterminate Western blot pattern: epidemiological determinants and PCR results in central African inhabitants. J Clin Microbiol 50:1663–1672. doi:10.1128/JCM.06540-11 22403426 PMC3347141

[35] Mahieux R, Horal P, Mauclère P, Mercereau-Puijalon O, Guillotte M, Meertens L, Murphy E, Gessain A. 2000. Human T-cell lymphotropic virus type 1 gag indeterminate western blot patterns in Central Africa: relationship to *Plasmodium falciparum* infection. J Clin Microbiol 38:4049–4057. doi:10.1128/JCM.38.11.4049-4057.2000 11060067 PMC87540

[36] Kazanji M, Mouinga-Ondémé A, Lekana-Douki-Etenna S, Caron M, Makuwa M, Mahieux R, Gessain A. 2015. Origin of HTLV-1 in hunters of nonhuman primates in Central Africa. J Infect Dis 211:361–365. doi:10.1093/infdis/jiu464 25147276

[37] Calattini S, Betsem E, Bassot S, Chevalier SA, Tortevoye P, Njouom R, Mahieux R, Froment A, Gessain A. 2011. Multiple retroviral infection by HTLV type 1, 2, 3 and simian foamy virus in a family of Pygmies from Cameroon. Virology 410:48–55. doi:10.1016/j.virol.2010.10.025 21087785

[38] Afonso PV, Cassar O, Gessain A. 2019. Molecular epidemiology, genetic variability and evolution of HTLV-1 with special emphasis on African genotypes. Retrovirology 16:39. doi:10.1186/s12977-019-0504-z 31842895 PMC6916231

