# Supplementary material for: High prevalence of HTLV-1 among blood donors in southeastern Gabon (Central Africa): a major challenge for transfusion safety in a semi-rural area
Source: Microbiol Spectr. 2026 Aug 6;14(9):e01755-26. doi: 10.1128/spectrum.01755-26 (PMC13532231; doi:10.1128/spectrum.01755-26)
Supplement: Supplementary file 1 [file spectrum.01755-26-s0002.docx]

**Supplemental figure 1. Flow chart of blood-donation records screened for HTLV-1**.
Duplicates were defined as multiple records for the same blood donors (confirmed) or near‑matches judged likely to be the same blood donors (probable). For repeat donors, we retained one donation with complete data; if multiple were complete, we retained the earliest donation.
